# Dynamic capabilities in tourism businesses: antecedents and outcomes

**DOI:** 10.1007/s11846-022-00567-z

**Published:** 2022-06-27

**Authors:** Hang T. T. Nguyen, Hanh Song Thi Pham, Susan Freeman

**Affiliations:** 1 Huddersfield Business School, University of Huddersfield, Queensgate, Huddersfield, HD1 3DH UK; 2 Leeds University Business School, University of Leeds, Leeds, LS2 9JT UK; 3 School of Management, Business School, University of South Australia, North Terrace, City West Campus, Adelaide, SA 5001 Australia

**Keywords:** Micro-foundations of dynamic capabilities, Dynamic capabilities view, Tourism services, Emerging economies, 00-02

## Abstract

This paper examines dynamic capabilities as a second-order construct and the antecedents and outcomes of dynamic capabilities. Analyzing data collected from 242 tourism firms in Vietnam, this paper finds a positive influence of firms’ human capital, organizational learning, environmental dynamism, and digital marketing on the dynamic capabilities. Dynamic capabilities have a significant and positive impact on these firms’ competitive advantage. The paper contributes to ongoing efforts to measure dynamic capabilities and explore the micro-foundations of dynamic capabilities.

**Supplementary Information:**

The online version contains supplementary material available at 10.1007/s11846-022-00567-z.

## Introduction

Dynamic capabilities refer to the ability of an organization to sense changes and seize opportunities, greater risk awareness, and the ability to take appropriate actions, make changes, and reconfigure and re-adjust the current business structure, operations, and routines (Michaelis et al. 2020; Nyamrunda and Freeman 2021; Teece et al. 1997). Despite a large amount of literature on dynamic capabilities conducted for more than two decades, empirical evidence is confined within manufacturing or innovation industries where the extensive application of advanced technology and rapid changes in technology are widely accepted as crucial features (Easterby-Smith et al. 2009; Jiang et al. 2019). Notably, research on dynamic capabilities in the service industries, especially in tourism sector, is limited. Meanwhile, tourism is considered a promising area in the context of economic development, environmental and socio-cultural changes, employment opportunities, new consumer values, the spread of technical knowledge, and the development of new markets and products (Dogru and Bulut 2018; Webster and Ivanov 2014). Businesses in the tourism industry need even more research attention, now that they are facing the challenges and disruptions the COVID-19 pandemic poses for the industry globally.

According to Teece and Pisano (1994), dynamic capabilities often arise in a fast-changing environment; it is, therefore, a highly contextual and contingent concept. This means that such capabilities can be understood better if they are linked to the business environment in which they take place. For example, in the manufacturing industry, especially the high-end technology-related industries, innovation is faster and more visible than in service industries (Snyder et al. 2016; Taques et al. 2020). Therefore, research on dynamic capabilities is conducted primarily in manufacturing enterprises where the process, output, and operations are more easily identifiable as dynamic capabilities (den Hertog et al. 2010). However, the results drawn from manufacturing industries may not be applicable to the service sector in general and the tourism sector specifically. This is because of significant differences between the manufacturing and service industries, such as the greater need for manufacturers to buy and maintain physical assets and inventory or the discrepancies in managing internal projects to address the growing needs of customers and suppliers (Biesenthal et al. 2019). Despite evidence of innovation embedded in the processes and procedures offered to customers in service delivery (Gustafsson et al. 2020; Taques et al. 2020), the service sector remains under-researched.

This research, therefore, aims to gain new insights into the understanding of dynamic capabilities in the service industries and, more specifically, in the tourism sector. Accordingly, this study considers the following research question: *How can dynamic capabilities be measured in tourism firms, and what are the determinants and outcomes of dynamic capabilities?*

This research makes two main contributions to the dynamic capabilities literature. First, this study improves the knowledge regarding the measurement of dynamic capabilities. Such capabilities are not easy to quantify (Barrales-Molina et al. 2014). The debate on how to measure them is ongoing (Biesenthal et al. 2019; Laaksonen and Peltoniemi 2018). Adapted from the dynamic capabilities construct suggested in Fainshmidt and Frazier’s (2017) and Wilden et al.’s (2013) studies of different industries, this research has modified and validated the measurement of the *second-order dynamic capabilities construct for tourism businesses*. While other studies have, for methodological reasons, measured the different clusters of capabilities that belong to dynamic capabilities as first-order constructs, a second-order model helps to explain better the covariance more parsimoniously with fewer parameters (Chen et al. 2005; Rindskopf and Rose 1988). Given the specific features of the tourism sector in terms of the scope and activities inherent within each cluster of dynamic capabilities and the original dynamic capabilities definition by Teece et al. (1997), a modified second-order construct is needed to test whether dynamic capabilities are covert in tourism firms and how best to reflect the features of such capabilities. We argue that with three group parameters corresponding to sensing, seizing, and reconfiguring, it is better for these capabilities to covary directly with dynamic capabilities and better reflect the nature of dynamic capabilities defined by Teece et al. (1997). Moreover, this second-order construct further reduces the number of first-order constructs in the main research model, improving the precision of the measurement model while still reflecting the nature of the dynamic capabilities view.

Second, this research is one of few pioneering studies of the dynamic capabilities at the firm level in the tourism sector of an emerging economy. Although much important work has been carried out on examining dynamic capabilities in hospitality settings, such as Pattanasing et al. (2021) and Krupskyi and Grynko (2018), several questions remain for the rest of the tourism sector. For example, a study by Nieves and Haller (2014) examines dynamic capabilities in the Italian hotel sector. However, to the authors' knowledge, no study has been conducted in the broader tourism industry. Therefore, this study is the first to provide a comprehensive view of dynamic capabilities and the different determinants as micro-foundations for dynamic capabilities in tourism firms.

We first discuss the theoretical background for dynamic capabilities and the different antecedents and outcomes. We then outline the quantitative design and methods to measure dynamic capabilities and test hypotheses empirically. Next, we present the empirical results and discuss the findings. Finally, we offer theoretical contributions, address the study’s limitations, and provide future research directions and managerial implications.

## Theoretical background

### Dynamic capabilities

‘Dynamic capabilities’ are defined in the literature in various ways. Some scholars perceive them as underpinning processes and routines that facilitate the development of firms (Eisenhardt and Martin 2000; Winter 2003; Zott 2003). Others view operational issues as existing at multiple levels of the organization, such as operating portfolios of projects which require several operational capabilities to achieve short-term and long-term objectives (Dasari et al. 2015). Some construe them as activities (Ali et al. 2012; Teece 2014). A further group of scholars views dynamic capabilities as resources, assets, or the organization itself. In this study, we agree with Teece’s (2018) view regarding the nature of dynamic capabilities. Teece (2018) contends they are not resources, which thus need to be distinguished from dynamic capabilities even though the dynamic capabilities view (DCV) is an extension of the resource-based view (RBV). This view avoids confusion with the organizational assets or resources that form the core of dynamic capabilities. Our study adopts the definition of dynamic capabilities originally suggested by Teece et al. (1997, p. 516) as “the firm’s ability to integrate, build, and reconfigure internal and external competencies to address rapidly changing environments.”

In tourism research, DCV is applied as the theoretical framework in a number of research projects. Most research focuses on the hotel sector (Fraj et al. 2015; Leonidou et al. 2015; Marco-Lajara et al. 2017). Others focus on tourism innovation (Sainaghi et al. 2017; Verreynne et al. 2019) and competitive advantage (Evans 2016; Nieves and Haller 2014), especially eco (Leonidou et al. 2015) and green competitive advantage and management (Mittal and Dhar 2016) in tourism SMEs (Brida et al. 2016). Most research utilizes DCV as a theoretical lens to view and explain competitive advantage, the sustainability of competitive advantage, performance, or firms' innovation. As such, little research analyzes how dynamic capabilities and their micro-foundations are manifested in tourism firms.

Dynamic capabilities can be viewed as a combination of all capabilities relating to sensing, seizing, and reconfiguring/transforming and not as a single independent capability of each (Nyamrunda and Freeman 2021). According to Byrne (2010), the higher-order model represents a seemingly distinct hypothesis, and associated constructs can be gauged by one or more common underlying higher-order construct(s). Furthermore, the second-order factor model is more parsimonious and provides error-free estimates of both general and specific factors (Chen et al. 2005). Therefore, we argue that a second-order (higher-order) factor model is more precise than a first-order model in measuring dynamic capabilities. In addition, the higher-order three-factor model of dynamic capabilities provides insights into how the three first-order factors contribute to overall dynamic capabilities.

### The antecedents of dynamic capabilities

#### Human capital

According to the RBV, physical, human and organizational assets can be used to implement value-creating strategies (Barney 1991). These resources can be configured and reconfigured in ways that cannot be matched or imitated easily by competitors (Barney 1991; 2001). The relationship between an organization's human capital and dynamic capabilities is described as micro-foundational (Nyamrunda and Freeman 2021).

Human capital refers to the knowledge, skills, and attributes embodied in individuals that can be used to yield professional services (Coppin 2017; Pennings and Lee 1998). The micro-foundational literature on the influence of human capital on the operation of organizations highlights that firms with employees of high levels of knowledge and experience will have more capabilities to identify the resource base and will understand the requirements to execute the alterations needed to better cope with an ever-changing environment (Gerrard and Lockett 2018; Kallmuenzer et al. 2019; Nyamrunda and Freeman 2021). Employees' implicit and explicit knowledge will determine an organization’s ability to solve problems or create new knowledge (Cross and Baird 2000; Salvato and Vassolo 2018). Augier and Teece (2009) argue that a firm's success is dependent on having highly skilled employees with abilities to harmonize, unite, and incorporate the firm’s resources. Hence, the role of people in the organization as the determinant of dynamic capabilities should be taken into account (Singh and Rao 2016). Rothaermel and Hess (2007) find that human intellectual capital underpins the building of dynamic capabilities that enable firms to adapt to radical technological changes. Nyamrunda and Freeman (2021) go further by developing a conceptual model that shows how strategic sensitivity, resource fluidity, and leadership unity entrenched in micro-foundational activities influence the relational dimensions (i.e., communication, social bonds, and knowledge), which builds trust in small business cross-border buyer–seller relationships to sustain dynamic relational capability.

The arguments therefore indicate that a higher level of knowledge, skills, and experiences endows individuals with an exceptional ability needed to acquire and apply new and valuable knowledge. It thus encourages the renewal of a firm's resource base. Accordingly, the following hypothesis is proposed:

##### H1

Human capital directly and positively impacts the dynamic capabilities of tourism firms.

#### Organizational learning

An organizational learning culture refers to “one in which learning is recognized as absolutely critical for business success; in such an organization, learning has become a habitual and integrated part of all organizational functions” (Marquardt 2002, p. 27). From a strategic perspective, the development of an organizational learning culture should, ideally, start with every member of the organization and then spread to the whole organization until it is embedded in the organizational structure, processes, and routines (Cheung and Zhang 2020; Hirst et al. 2009). Learning is a particular type of process that is fundamental to the growth and evolution of dynamic capabilities (Bowman and Ambrosini 2003; Eisenhardt and Martin 2000; Zollo and Winter 2002). Other scholars like Winter (2003) and Easterby-Smith and Prieto (2008) state that the learning process enables the creation and modification of dynamic capabilities. Therefore, it is reasonable to propose that organizational learning affects the development of dynamic capabilities in firms*.* Moreover, organizations with a better learning organizational culture have better learning mechanisms, and organizational members are more able to embrace, learn, and practice new knowledge (Day 1994; Huber 1991).

It is suggested that organizational learning supports dynamic capability. An organization’s core capabilities are interwoven with the organizational learning process (Ciborra and Andreu 2001; Pu and Soh 2018). It is argued that dynamic capabilities are influenced by organizational learning mechanisms, including knowledge accumulation, articulation, codification, and the learning culture (Zollo and Winter 2002)*.* A study by Hung et al. (2010) found that dynamic capabilities mediate the relationship between organizational learning culture and organizational performance. In our study, however, we consider organizational learning culture to be one of the determinants that influence the level of dynamic capabilities in the organization. The following hypothesis is therefore proposed:

##### H2

Organizational learning culture has a direct and positive impact on the dynamic capabilities of tourism firms.

#### Digital marketing

Digital marketing refers to leveraging the unique capabilities of new interactive media to produce new forms of interactions and connections between consumers and marketers (Chaffey and Ellis-Chadwick 2019). It also refers to incorporating interactive media with the different components of the marketing mix (Kannan and Li 2017; Parsons et al. 1998). A study by Tallon and Pinsonneault (2011) identifies the mediating role of firm agility on the impact of strategic information technology alignment on business performance. Dynamic capabilities are considered integral in transforming external technologies into firms’ renewed technological resources (Abrate et al. 2020; Li-Ying et al. 2016).

Internet technology has impelled many entrepreneurs to participate in a thorough evaluation of how their firms assemble, integrate, employ, and distribute information to customers, employees, and supplier networks whilst continuing innovative in their capacity to generate business models and deliver value effectively and efficiently to all (Guo et al. 2018; Hunt and Madhavaram 2019; Kraus et al. 2019). All these activities are part of the dynamic capabilities that organizations possess. More specifically, Roberts and Grover (2012) find that firm agility (i.e., customer sensing and responding capabilities) influences firm performance, but in different ways. While customer sensing capability positively influences firm performance, customer responding capability has the reverse impact. Furthermore, recent digital technology development as a result of the Covid-19 pandemic and interruptions to supply chains has helped firms put dynamic capabilities into practice more readily than ever before, allowing more efficient collaborations and interactions between organizations and their stakeholders (Coreynen et al. 2020; Warner and Wäger 2019). Such efficient collaborations also help organizations facilitate new types of innovation, shape more strategies, reach more customers, and extend organizations' networks (Sambamurthy et al. 2003).

In addition, evidence from the literature below shows that digital technology is beneficial for all sensing, seizing, and reconfiguring activities. Digital technology application in marketing activities, such as Web services, data warehousing, digital market intelligence, or customer relationship management, helps leverage dynamic capabilities (El Sawy et al. 2010; Overby et al. 2006; Zhang et al. 2013). With the support of digital technology, organizations can perform transactions, exchange information, and facilitate real-time integration with customers and suppliers more quickly and continuously. Consequently, more innovative digital products and services can be generated (Wheeler 2002). Regarding sensing capabilities, digital technology provides tools for organizations to obtain market intelligence by analyzing customer perceptions and communicating with prospective customers (Frasquet et al. 2013). As for seizing capabilities, digital platforms support the information flows between different stakeholders and enable firms to share and stream their complex processes (Rai et al. 2006). Finally, for reconfiguring capabilities, digital tools help firms to produce more abundant tacit knowledge, coordinate diverse knowledge management activities between geographically dispersed individuals, allow the precise replication of specific tasks with workflow arrangements with a reduced number of mistakes, and advance the prototyping process (Matarazzo et al. 2021; Vaccaro et al. 2009).

Hence, this research proposes the following.

##### H3

The application of digital marketing has a direct and positive impact on the dynamic capabilities of tourism firms.

#### Environmental dynamism

To identify environmental dynamism, this research refers to the concept developed by Miller and Friesen (1983) in considering three factors. The first dimension is the industry's rate of change and innovation and the unpredictability and uncertainty of competitors’ and customers’ actions (Schilke 2014). The second dimension is the ‘hostility’ or level of threat posed by the industry, and the final dimension is the heterogeneity or level of complexity of the organization’s target markets (Azadegan et al. 2013)*.* Environmental dynamism is thus considered to be: (1) the rate of change and innovation in the sector and the unpredictability and uncertainty of competitors and customers’ actions; (2) the level of threat posed by the industry; and (3) the level of complexity of the target markets (Mikalef et al. 2020). Environmental dynamism is a causal condition for dynamic capabilities (Gelhard et al. 2016). The influence of dynamic capabilities depends on external settings (Eisenhardt and Martin 2000) and equates with the dynamism of the environmental conditions (Gelhard et al. 2016; Li and Liu 2014).

Following this line of argument, the level of external dynamism directly influences the strategies adopted by firms. It is influential in reconfiguring directions, business models, and routines in order to adapt to changes in the industry, targeted markets, competitors, policies, or technology (Warner and Wäger 2019; Wilden et al. 2013). Therefore, it is imperative to examine the effect of external dynamism on the dynamic capabilities of organizations. Given that firms in the tourism sector are affected “very quickly by environmental variables, changes in customer preferences, high competition, etc.,” it is essential to “examine the effects of obtaining, transforming, exploiting, and using external information on firm performance” (Kale et al. 2019, p. 281). The recent shock and disruptions caused by the COVID-19 pandemic to businesses have pointed to the importance of external dynamism in prompting the formation dynamic capabilities of an organization and, therefore, the necessary capabilities to respond to a fast-changing environment quickly (Breier et al. 2021; Jiang and Wen 2020). It is hence proposed that:

##### H4

External dynamism factors have a direct and positive influence on the dynamic capabilities of tourism firms.

### The outcomes of dynamic capabilities–competitive advantage

Discussions on the outcomes of dynamic capabilities focus on improving organizational performance (Augier and Teece 2009; Wilden et al. 2013). When evaluating this relationship to performance, numerous criteria and indexes can be used, of which the two most common are the financial performance and strategic performance of organizations in gaining a desirable position in markets to achieve the set goals. Competitive advantage is regarded as a “superior market position” (Weerawardena 2003, p. 21) that organizations possess to provide the market with superior products or services while attaining market dominance (Hunt and Morgan 1995). Previously, a firm’s primary objective was to achieve superior financial performance. Financial indicators were considered the sole and vital measures of an organization’s performance (Barney 1991). Financial performance is often specified by indicators such as profits and return on investment (Hunt and Morgan 1995). However, the firm's success cannot rely on using exceptional financial performance as a sole indicator of competitive advantage (Barney 1991; Day and Wensley 1988). From an RBV, the organization attains superior financial performance when it has superior skills or resources (Arbelo et al. 2020). This results in positional advantages and superior performance outcomes regarding relative profits and market share (Seyoum 2020).

Consequently, by nature, competition involves constant attempts by firms to obtain a comparative advantage in the resources they possess, which will yield a marketplace position of competitive advantage (Hunt and Morgan 1996). This will result in superior financial performance (Hunt and Morgan 1995). Porter (1991, p. 96) argues that a firm succeeds when it gains a “superior and sustainable performance… relative to the world’s best rivals”. Therefore, in evaluating the competitive position of an organization, it is necessary to look at both financial performance and marketplace position. This study thus defines ‘competitive advantage’ as the ‘superior market position’ an organization possesses to provide the market with superior products and services. These superior products and services then result in a positional advantage and superior performance outcomes with respect to profits and market dominance (Hunt and Morgan 1995; Jantunen et al. 2018; Porter 1991).

Research on the tourism industry discusses competitive advantage from the perspective of the RBV (Liu 2017; Molina-Azorin et al. 2010). The different resources for competitive advantage from the RBV include knowledge (Fraj et al. 2015; Thomas and Wood 2014), leadership capabilities (Sainaghi et al. 2017), innovation capabilities (Kale et al. 2019), and/or location (Denicolai et al. 2010; Molina-Azorin et al. 2010). Recent research by Verreynne et al. (2019) focuses more heavily on green competitive advantage and sustainable competitive advantage. There is a strong and compelling argument by Evans (2016) on how to facilitate innovation for sustainable competitive advantage.

Dynamic capabilities are important for tourism firms because the industry is now facing changes in all aspects of the service process, with rapid technological digitalization as the main factor. Some examples of technological advancements that are revolutionizing the industry are mobile technology, big data, the Internet of Things, blockchain, 5G technology, and augmented reality, to name just a few. The transformation that tourism companies need to focus on is not merely on changing the travellers’ experience but also on reforming the whole system, leading to significant innovation or even the creation of new business models. Such changes in technology and market trends require companies operating in the tourism industry to develop the dynamic capabilities to rapidly identify, transform, reform business processes, or create new operation models. All these activities will require ordinary capabilities needed for daily operations and using existing resources (Qaiyum and Wang 2018; Schriber and Löwstedt 2020) and superior capabilities such as dynamic capabilities to identify resources to execute the innovative initiatives at a higher level. As such, Nieves et al. (2016) argue that innovation in tourism services is based on capabilities for developing knowledge and learning and that the dynamic capabilities approach is a valuable framework for investigating innovation in the industry (Camisón and Monfort-Mir 2012). Innovation in tourism is also suggested on the basis of service design and service processes, rather than merely product innovation (Gomezelj 2016).

By reconfiguring current resources, organizations that possess dynamic capabilities will have a more positive impact on the firm's competitive advantage (Eisenhardt and Martin 2000; Teece and Pisano 1994). In a study by Schilke (2014), the influence of dynamic capabilities on competitive advantage was found to be contingent on the level of external dynamism. Dynamic capabilities are considered key to acquiring a competitive advantage in strategic management (Agwunobi and Osborne 2016; Li and Liu 2014). They are also the critical antecedents driving the innovation-based competitive advantage process (Chatzoglou and Chatzoudes 2018; Muhic and Bengtsson 2019; Salunke et al. 2019), determinants for achieving a competitive advantage (Nieves and Haller 2014), and a handy tool for organizations to gain a competitive advantage, even in a highly volatile environment (Monteiro et al. 2019; Schwarz et al. 2020). Therefore, the following hypothesis is proposed:

#### H5

Dynamic capabilities have a direct and positive impact on the competitive advantage of tourism firms.

**Fig. 1:**
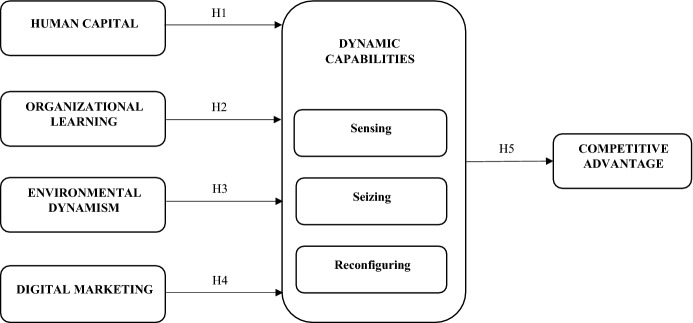
The antecedents and outcome of dynamic capabilities in tourism businesses

Figure 1 summarises the relationships between dynamic capabilities and their antecedents and outcomes.

## Method

### The research context

Vietnam is used as the research context for this paper. It is one of Southeast Asia’s most picturesque countries, attracting an increasing number of travelers to its lush mountains, dynamic cities, and sandy beaches before COVID-19. Tourism in Vietnam has a vast potential for development, and it remains an attractive destination despite the pandemic challenges.^1^ The *World Economic Forum* (2019) places Vietnam 63rd among 140 economies for tourism competitiveness, one of the top ten most improved economies, and the fastest improving nation in the Association of South East Asian Nations (ASEAN) (Calderwood and Soshkin 2019). According to the *World Economic Forum* (2017), Vietnam is predicted to be one of the top ten destinations for travel from 2016 to 2026 (Crotti and Misrahi 2017). These figures demonstrate the rapid and dynamic growth of the tourism sector in Vietnam and the opportunities it provides.

### The sample and data collection

The research population was determined as Vietnamese domestic tourism companies. According to official figures from the Vietnam National Administration of Tourism, in 2017, there were 824 hospitality businesses from *3 stars* and above and 1430 tourism companies nationwide (VNAT 2018). The sample size was determined as per the rule of thumb suggested in multivariate analysis literature (Hair et al. 2014). Specifically, there are 42 items developed for our measurement model. According to the criterion suggested by Hair et al. (2014) for choosing the expected sample size, a subjects-to-items ratio should be from 5 to 20 to ensure possible identification of the proposed statistical model. This means a sample N ranging from 210 to 840 was acceptable for running CFA and SEM in this study. Given the time constraints and financial resources for data collection, 1,000 questionnaires were distributed with a usable target response rate of at least 21% (to yield at least N = 210). After the sampling criteria, target population and sample were identified, this research utilized different sampling methods to optimize the response rate.

The sampling techniques used to collect data consist of convenient sampling, stratified sampling, and snowball sampling to represent different types of businesses in the tourism sector and represent the three key regions of Vietnam (the North, the Centre, and the South). The data collection was undertaken in three stages. The first stage utilized convenient sampling conducted at the national and international tourism fairs in Vietnam's two biggest cities: Ho Chi Minh City (in March 2017) and Hanoi City (in April 2017). This technique generated 112 responses from both events.

The second stage involved stratified sampling whereby the number and contact details of travel and hospitality businesses were taken from the Vietnam National Agency for Tourism (VNAT) statistics section. The percentage of each province’s businesses over the total national number (1,578 travel companies and 1,577 hospitality addresses across 63 provinces) was calculated, and the subsequent numbers of tourism firms required in those provinces were determined. Resource constraints meant that half this number would be required. The calculation showed that 500 questionnaires should be distributed to 53 representative provinces (as there are ten provinces with less than 0%). Therefore, 500 pre-paid postal questionnaires were sent and administered from March to May 2017. This technique did not generate a reasonable response rate even with follow-up calls where possible. Only 10 responses were returned out of 500 distributed questionnaires.

During the first and second stages of sampling, it became apparent that the response process was prolonged and limited. Therefore, we decided to initiate the third stage combined with these two stages. Snowball sampling was employed based on our professional contacts from colleagues, friends, and relatives who recommended managers/directors in tourism firms they knew personally. Therefore, unused questionnaires from convenience sampling were used for the snowball samples. After a respondent completed a questionnaire, we asked if they knew someone who could answer the questionnaire like what they had done. This tactic worked well, and 125 responses were generated using this sampling technique.

In total, 247 responses were received using all three sampling techniques. A paper questionnaire was primarily used unless participants requested an online version for their convenience, in which case they were sent the same version created on Google. This approach ensured the questionnaire was sent to the right respondents and not to online groups and forums where we could not control the number of questionnaires distributed and thus calculate the response rate. Overall, 247 responses to 1,000 disseminated questionnaires meant the response rate was 24.7%.

To ensure the integrity of the research and the voluntary nature of the participation, we did not give financial incentives to the respondents to respond to the survey. After deleting three responses with a missing (incomplete) rate of more than 30% and two unengaged responses, 242 were deemed usable. This represented a response rate of 24.2%, within the acceptable response rate (13.8–56.2%) for academic research on organizations (Baruch and Holtom 2008).

#### Respondent selection

The ideal informants in this study were deemed to hold the position of deputy head of a department and above in the business's management team. These key informants are argued to have experience and access to important information regarding the operation of the business and possess specialized knowledge of the tourism sector.

Given these considerations, the key informants selected for this study were senior and accessible informants across the organizations. They were also considered more reliable and provided standardized information given their seniority (Marshal 1996). Although multiple respondents may reduce the common method variance (Malhotra et al. 2017), it was not strictly conducted in this study as numerous businesses are small entities with fewer than five members in their organization. In such cases, one key informant was sufficient. Furthermore, because the key informants were asked to assess relationships between phenomena in the organization rather than the organization per se, they were qualified to evaluate these relationships with a high degree of accuracy and reliability.

### Measures

Twelve items from the original scales adapted from previous studies were removed in this research because of low factor loadings (less than 0.6), of large standardized residual covariance (larger than |2.58|) (Byrne 2010, p. 77), and the high correlation with other indicators simultaneously. Details of the removed items and reasons for such removal are in Supplementary Information 1.

#### Dynamic capabilities

Based on the definition suggested by Teece (2007, 2012) and Teece et al. (1997), dynamic capabilities in this study comprise three clusters of capabilities: sensing, seizing, and reconfiguring.

##### Sensing

Sensing measures the extent to which organizations sense changes (market changes, policy changes, technology changes, competitor changes, customer changes) in the internal and external environments. This study specifically measures the extent to which tourism firms respond to macro and micro changes that influence the operation of the business. It denotes an ability to carry out internal scanning to identify changes that businesses need to address. According to Nieves and Haller (2014), sensing capabilities is an endogenous variable that consists of four items.

##### Seizing

Seizing refers to the ability of organizations to capture efficiently and effectively, the opportunities identified through sensing by taking advantage of current tangible and intangible resources, routines, processes, and assets. The tool for measuring seizing was adapted from Fainshmidt and Frazier (2017), which was originally taken from Wilden et al. (2013).

##### Reconfiguring

Reconfiguring refers to the ability to continuously renew or reconfigure the current state of firms. The measurement for reconfiguring capabilities was adapted from Fainshmidt and Frazier (2017), where reconfiguring is also an endogenous variable.

According to Byrne (2010), second-order CFA is a statistical method utilized to confirm the theorized construct in a study loads onto a certain number of underlying sub-constructs or components*.* Therefore, using a second-order factor model is more appropriate than a first-order model. This is because the second-order model represents a seemingly distinct hypothesis, and associated constructs can be gauged by one or more common underlying second-order constructs (Byrne 2010). Moreover, the second-order three-factor model of dynamic capabilities explains how the three first-order factors contribute to overall dynamic capabilities.

#### Human capital

The measures were adapted from Nieves and Haller (2014), albeit with some modifications in wording and the removal of one item as recommended by the experts. These experts are practitioners and researchers in Vietnamese tourism. The removed item was ‘Our employees are widely considered the best in our industry’. It was eliminated because both experts said it was difficult for management to evaluate the phrase 'in our industry'. Even though all businesses are in the same industry, each organization operates within a small and different segment of the large tourism industry, such as in budget tours, luxury tours, hospitality, and accommodation, among others. Therefore, it would be impossible for the management to evaluate their employees without being biased. In the study by Nieves and Haller (2014), human capital is confirmed to positively affect the development of dynamic capabilities such as sensing, learning, integration, and coordination. The skills, knowledge, abilities, and experience of people in an organization are vital for the effective and efficient operation of the business. Such qualities, embedded in people’s thoughts and actions, are the intangible capital from which organizations can benefit.

#### Organizational learning

Questions on organizational learning culture were adopted partly from the Dimensions of Learning Organization Questionnaire (DLOQ) designed by Marsick and Watkins (2003). In their research, Marsick and Watkins (2003) measured organizational learning at three levels: individual level, team or group level, and organizational level. Although the items are comprehensive, it was impossible to adopt all 43 statements in this study. Therefore, based on a study by Hung et al. (2010, p. 292), we selected six key statements to describe the learning culture at an organizational level. The reliability of learning as an organizational level construct was confirmed in Hung et al.’s (2010) study as 0.88.

#### Digital marketing

This study adopted items relating to the two constructs (i.e. customer-related marketing activities, field-sales and channel member-related marketing activities) developed by Prasad et al. (2001, p. 106) because the other two constructs (i.e. marketing research-related and management communication activities) have not been well applied in Vietnam and were not recommended by the industry experts following an in-depth questionnaire discussion. Therefore, the final items comprised four customer-related marketing items (the first four items), four field-sales items, and channel member-related marketing activities (the last four items).

#### Environmental dynamism

The items for environmental dynamism are developed by Jansen et al. (2006) to measure the extent to which an organization’s external environment is characterized by harsh competition, demonstrated in the number of rivals and areas in which there is competition. This variable is empirically tested by Gelhard et al. (2016) as a mediator between dynamic capabilities and strategic performance.

#### Competitive advantage

Competitive advantage in this study is a construct comprising two dimensions of strategic performance (qualitative dimension), measured by the first three items, and financial performance (quantitative dimension), measured by the last three items (Fainshmidt and Frazier 2017; Schilke 2014). Both strategic and financial performance was measured in comparison to the competition. As cited in Schilke (2014, p. 188), these two performance dimensions were adapted from Jap (1999) and Weerawardena (2003).

All items were quantified by a 7-point Likert scale in which ‘0’ meant ‘strongly disagree’ and ‘6’ meant ‘strongly agree’. Respondents were key informants who held a management position in the organizations and were directly involved in the decision–making process.

### Questionnaire formation

The questionnaire was first prepared in English. To ensure the measures in both the source (English) and target (Vietnamese) versions were similar, standard translation and backward translation procedures were applied (Brislin 1970). The final English version was translated into Vietnamese (by a qualified translator). The Vietnamese version was then back-translated by other suitably qualified and experienced researchers working in strategic management and tourism management disciplines. Most of the corrections made were related to enhancing the explanations of items and further clarification in the introduction to the questionnaire. After correcting and clarifying the changes, another Vietnamese version was reviewed and refined to modify the wording to make it less technical and more understandable to potential Vietnamese respondents. The questionnaire was then sent to nine senior Vietnamese researchers in the UK and Vietnam to check whether further changes were required.

Before commencing the data collection, the questionnaire was sent to three experts working in the tourism sector to assess whether there were any possible misunderstandings and lack of coherence in the terminologies used in the industry. Each of these experts had over 15 years of experience working in the tourism sector and tourism training. They suggested that greater clarification was required to make the questionnaire more specific to the tourism industry. For example, it was agreed that ‘service’ should be clarified as a ‘tourism service’ so that respondents could quickly grasp the question's meaning. The final questions used for a survey are presented in Appendix 6..

## Data analysis and results

### Sample characteristics and measurement model

General information on the organizations studied is presented in Table 1.

**Table 1:** Sample profile

	Category	Frequency	Percent (100%)
Company age	≤ 5 years	118	48.8
	< 5 ≤ 10 years	54	22.3
	> 10 years	64	26.4
	Missing	6	2.5
Total capital (billion VND)	≤ 10 billion	126	52.1
	< 10 ≤ 50 billion	68	28.1
	> 50 billion	46	19
	Missing	2	.8
Employee number	≤ 50	158	65.3
	< 50 ≤ 100	32	13.2
	> 100	40	16.5
	Missing	12	5
Location	North	166	68
	Central	30	12.4
	South	41	16.9
	Missing	5	2.1
Business activities	Single service^a^	28	11.6
	Mixed services^b^	214	88.4

^a^The business provides only one type of service such as tour operator, travel agency, tourism transport provider, accommodation, food and beverage services, cultural and recreational services

^b^The business provides different services such as tour operator, travel agency, tourism transport provider, accommodation, food and beverage services, cultural and recreational services. The common feature of tourism businesses is that they do not operate solely in one sub-sector of tourism but engage in numerous other integrated and combined activities related to other sub-sectors such as transport for travel, recreational activities, food and beverage, and accommodation

We validated the measurement models by performing CFA for six constructs. Items were restricted to load only onto their priori specified factor and were allowed to correlate with one another. We refined the measurement model by removing indicators with factor loadings lower than 0.6 and then re-running the CFA.^2^ A summary of the average variance extracted and the construct reliabilities of the final measurement model is presented in Table 2. The overall fitness indices suggest a good fit for the measurement model (χ^2^ = 569.869; df = 383; *p* = 0.000; χ^2^/df = 1.558; CFI = 0.94; TLI = 0.93; RMSEA = 0.048). Each item significantly loads onto its respective construct (*p* < 0.001) with values ranging from 0.64 to 0.89. Each construct has high composite reliability (ranging from 0.76 to 0.91), exceeding the usual 0.70 benchmarks (Hair et al. 2014). Convergent validity is satisfactory as the standardized loading for each item and the average variance extracted (AVE) both exceed the 0.5 thresholds recommended by (Hair et al. 2014). The internal consistency of the multi-item scales is also satisfactory as the composite reliability (CR) of each exceeds the 0.7 cut-off recommended by Hair et al. (2014). The details of the CFA model and results can be found in Supplementary Information 2.

**Table 2:** CFA results for all constructs

Constructs	Items	Factor loading	AVE	CR	MSV
Dynamic capabilities (DC)	SS1	0.651	0.58	0.81	0.50
	SS2	0.693			
	SS3	0.739			
	SS4	0.789			
	SZ1	0.698			
	SZ3	0.775			
	SZ4	0.639			
	RCFG2	0.752			
	RCFG3	0.771			
	RCFG4	0.838			
Human capital (HC)	HC1	0.687	0.57	0.84	0.41
	HC2	0.761			
	HC3	0.845			
	HC4	0.709			
Organizational learning (OL)	OL1	0.750	0.51	0.76	0.50
	OL2	0.700			
	OL4	0.698			
Environmental dynamics (ED)	ED2	0.741	0.57	0.80	0.43
	ED3	0.761			
	ED4	0.764			
Digital marketing (DM)	DM3	0.669	0.55	0.86	0.18
	DM4	0.672			
	DM5	0.811			
	DM6	0.826			
	DM8	0.714			
Competitive advantage (CA)	CA1	0.765	0.66	0.90	0.19
	CA2	0.861			
	CA3	0.886			
	CA4	0.720			
	CA6	0.802			

*AVE* Average variance extract (all above 0.5); *CR* Composite reliability (all above 0.7); *MSV* Maximum shared variance

Table 3 shows the correlation matrix and means for all constructs of the model.

**Table 3:** Correlation matrix and means for all constructs

Variables	Mean	CA	DC	DM	ED	OL	HC	RCFG	SZ	SS
Competitive Advantage	3.598	1								
Dynamic Capabilities	4.703	0.335	1							
Digital Marketing	4.615	0.16	0.423	1						
Environmental Dynamism	4.740	0.431	0.653	0.328	1					
Organizational Learning	4.873	0.327	0.709	0.399	0.63	1				
Human Capital	4.373	0.35	0.593	0.307	0.438	0.637	1			
Reconfiguring	4.440	0.258	0.769	0.325	0.502	0.545	0.456	1		
Seizing	4.464	0.255	0.762	0.322	0.497	0.54	0.452	0.586	1	
Sensing	5.155	0.252	0.752	0.318	0.491	0.533	0.446	0.578	0.573	1

We then examined the possibility of common methods bias following Podsakoff et al. (2003) by performing Harman’s one-factor test in two steps (Aguirre-Urreta and Hu 2019). First, all the variables were entered into an exploratory factor analysis. As a result, no single factor emerged that accounted for the majority of the variance (ranging from 28.63 to 50%). We, therefore, concluded that there is no common factor. Second the standardized regression weights (*β*) of the two models–with and without Common Latent Factor – were compared. If the differences between the two coefficients had been larger than 0.2, this might have indicated common method bias. However, the results suggested that common method bias was not a concern and was unlikely to confound the interpretations of our results. The matrix that indicates discriminant validity is presented in Table 4.

**Table 4:** Validity check

	CR	AVE	MSV	MaxR(H)	DC	HC	OL	ED	DM	CA
DC	0.805	0.579	0.503	0.805	**0.761**					
HC	0.839	0.567	0.406	0.908	0.593	**0.753**				
OL	0.760	0.513	0.503	0.929	0.709	0.637	**0.716**			
ED	0.799	0.571	0.426	0.945	0.653	0.438	0.630	**0.755**		
DM	0.858	0.550	0.179	0.960	0.423	0.307	0.399	0.328	**0.741**	
CA	0.904	0.655	0.186	0.972	0.335	0.350	0.327	0.431	0.160	**0.809**

*AVE* Average variance extract (all above 0.5); *CR* Composite reliability (all above 0.7); *DC* Dynamic capabilities; *CA* Competitive advantages; *HC* Human capital; *OL* Organizational learning; *ED* Environmental dynamism; *DM* Digital marketing; *MaxR(H)* Maximum reliability; *MSV* Maximum shared variance

### Hypothesis testing results

Table 5 presents the path analysis results using SEM on the whole sample. This shows our baseline model was a good fit (χ^2^ = 784.167; df = 393; *p* = 0.000; χ^2^/df = 1.995; CFI = 0.89; TLI = 0.88; RMSEA = 0.064).

**Table 5:** The regression path coefficient and its significance

Hypothesis				Standardized coefficients	Conclusion
H1 ( +)	Human capital Dynamic capabilities			0.354***	Supported
H2 ( +)	Organizational learning Dynamic capabilities			0.404***	Supported
H3 ( +)	Environmental dynamism Dynamic capabilities			0.447***	Supported
H4 ( +)	Digital marketing Dynamic capabilities			0.188**	Supported
H5 ( +)	Dynamic capabilities Competitive advantage			0.366***	Supported

***, **, and * denote *p* < 0.001, 0.01, and 0.05 respectively

The Chi-squared results (χ^2^/df = 1.995 < 3) and RMSEA (< 0.07) show a good fit. However, CFI and TLI were slightly below the suggested level of at least 0.92 with N < 250 and m ≥ 30 (Hair et al. 2014, p. 584), suggesting a mediocre fit. Theoretical support is required to specify the model and achieve an ideal fit. Furthermore, it is important that the model specifications best approximate the theory to be tested rather than increase model fit (Hair et al. 2014). Byrne (2010) also emphasizes that fit indexes do not reflect the plausibility of a model and that judgments do depend on researchers. We adopt Mai et al.’s (2021, p. 11) “best-to-fit-a-specific purpose thinking” while testing and evaluating the models to determine the best performing fit indicators and cut-off values.

As shown in Table 5, within the model the positive impacts of Human Capital (β = 0.354; *p* = 0.000), Organizational Learning (β = 0.404; *p* = 0.000), Environmental Dynamism (β = 0.447; *p* = 0.000), and Digital Marketing (β = 0.188; *p* = 0.017 < 0.05) were supported. Therefore, H1, H2, H3, and H4 are confirmed. Regarding the antecedents that influence Dynamic Capabilities, Environmental Dynamism has the most substantial influence (β = 0.447), followed by Organizational Learning (β = 0.404), Human Capital (β = 0.354), and Digital Marketing (β = 0.188).

The empirical results also provide statistical support for the positive impact of Dynamic Capabilities on Competitive Advantage (β = 0.366; *p* < 0.001). Thus, H5 is confirmed.

**Fig. 2:**
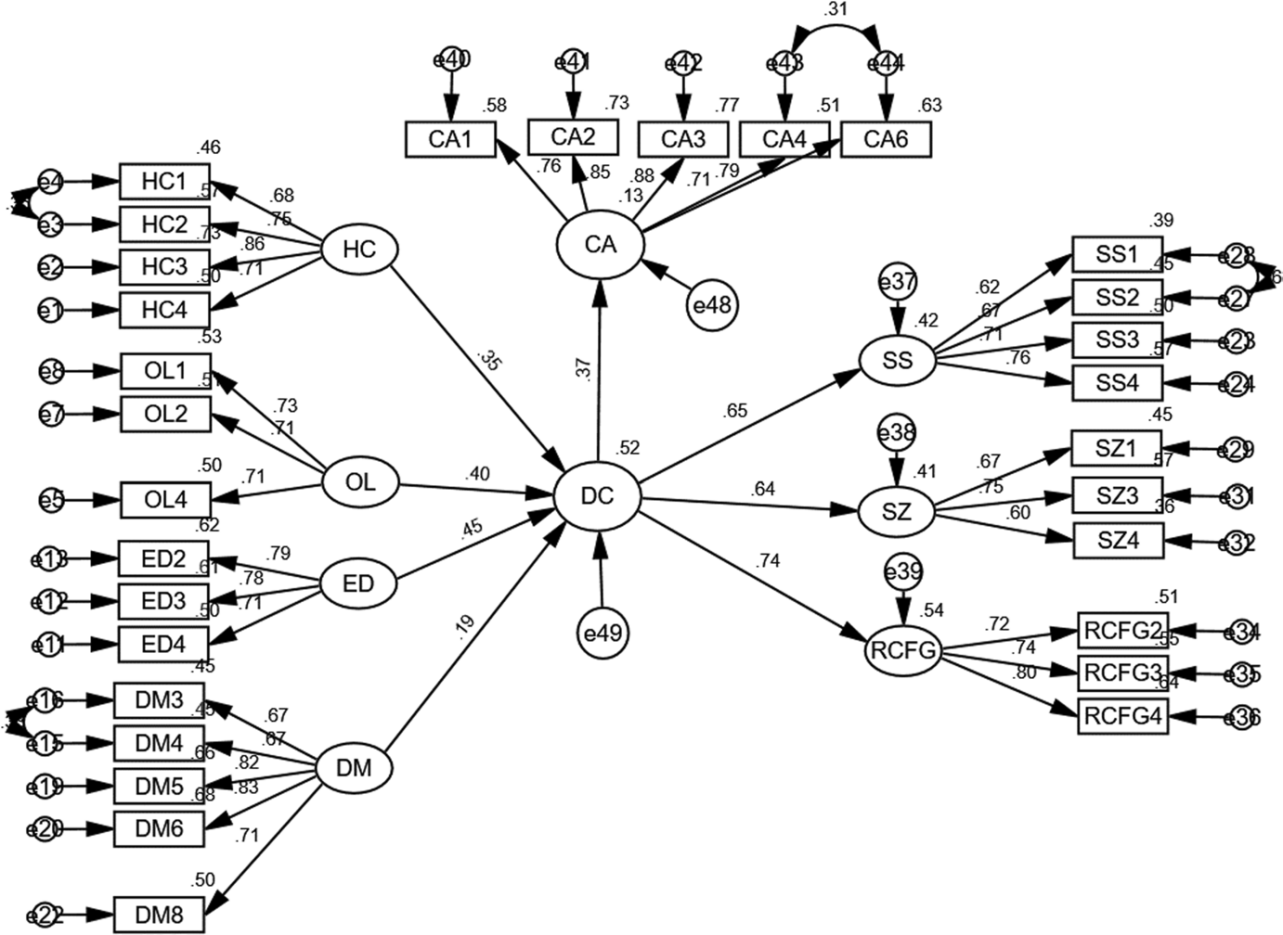
Structural Equation Modelling results

The full results of the baseline model are displayed in Fig. 2 below.

### Robustness check

For robustness check, we conducted a model variation test by selecting 200 cases out of the overall sample of 242. SEM was applied to a sub-sample of 200 initial observations from the entire sample, the results of which are reported in Table 5. The fit indices of the robust check model are also satisfactory (χ^2^ = 736.219; df = 393; *p* = 0.000; χ^2^/df = 1.873; CFI = 0.882; TLI = 0.869; RMSEA = 0.066). The results presented in Table 6 are consistent with those reported in Table 5, suggesting that our findings are robust.

**Table 6:** The regression path coefficient and its significance of the model with the first 200 cases

Hypothesis				Standardized coefficients	Conclusion
H1 ( +)	Human capital Dynamic capabilities			0.388*	Supported
H2 ( +)	Organizational learning Dynamic capabilities			0.389**	Supported
H3 ( +)	Environmental dynamism Dynamic capabilities			0.482***	Supported
H4 ( +)	Digital marketing Dynamic capabilities			0.219*	Supported
H5 ( +)	Dynamic capabilities Competitive advantage			0.410***	Supported

Note: ***, **, and * denote *p* < 0.001, 0.01, and 0.05 respectively

### Testing alternative models and mediation analysis

Alternative models were built by adding further direct paths between variables to assess whether each alternative model was better than the hypothesized model and whether the hypothesis test results held. Four additional paths were added, and the new models were run. The results are presented in Table 7.

**Table 7:** Results of testing alternative models with additional paths

Models	Model fit indices	Accept
Alternative model 1: direct path from HC CA	(χ^2^ = 823.470; df = 421; *p* = .000; χ^2^/df = 1.956; CFI = .888; TLI = .876; RMSEA = .063)	No
Alternative model 2: direct path from OL CA	(χ^2^ = 824.898; df = 421; *p* = .000; χ^2^/df = 1.959; CFI = .887; TLI = .875; RMSEA = .063)	No
Alternative model 3: direct path from ED CA	(χ^2^ = 819.560; df = 421; *p* = .000; χ^2^/df = 1.947; CFI = .889; TLI = .877; RMSEA = .063)	No
Alternative model 4: direct path from DM CA	(χ^2^ = 825.289; df = 421; *p* = .000; χ^2^/df = 1.960; CFI = .887; TLI = .875; RMSEA = .063)	No

*CA* Competitive advantages; *HC* Human capital; *OL* Organizational learning; *ED* Environmental dynamism; *DM* Digital marketing; *CFI* Comparative fit index; *df* degrees of freedom; *RMSEA* Root mean square error of approximation; *TLI* Tucker-Lewis index

Comparing the fitness of the alternative models with the baseline model (χ^2^ = 784.167; df = 393; *p* = 0.000; χ^2^/df = 1.995; CFI = 0.89; TLI = 0.88; RMSEA = 0.064), all the fitness indexes are somewhat equal to or below those of the baseline model and generate only a minimal difference. Hence, these additional paths and alternative models were not selected. Accordingly, it can be concluded that the current research model was the most appropriate and the hypothesis test results were robust.

Nevertheless, the alternative models still passed the acceptable threshold for the fitness indices and indicated the potential mediation effect of dynamic capabilities. We subsequently conducted further tests to determine whether dynamic capabilities mediate the relationships between antecedents (Human Capital, Organizational Learning, Environmental Dynamism, Digital Marketing) and the outcome (Competitive Advantage). Following Zhao et al.'s (2010) procedure, we used bootstrapping^3^ (with 5,000 resamples and 95% confidence intervals) of the direct and indirect effects in AMOS 21. The bootstrapping procedure provided associated p-values for each path.

The direct and total effects of each path are shown in Table 8. The results show that dynamic capabilities have a full mediation impact on the relationship between Digital Marketing and Competitive Advantage and direct-only non-mediation, in line with Zhao et al. (2010). Even though there is no indirect effect, this should not be viewed as a failure because a significant direct effect signifies undiscovered mediators (Zhao et al. 2010). This is because dynamic capabilities are a second-order construct that might weaken the impact of each subset of capabilities (sensing, seizing, and reconfiguring). We suggest that the mediation impact might be more apparent if each subset of dynamic capabilities is tested separately as a mediator in the relationships between antecedents and the outcome.

**Table 8:** Mediation results

Model 1 (no DC as a mediator)									Model 2 (with DC as a mediator)				
Paths	Direct effects (c’)	Path		Direct effects (a)	Paths	Direct effects (b)		Indirect effects (ab)	Total effects (c = c’ + ab)	VAF (ab/c)	95% CI bias-corrected bootstrap intervals)	*p*	Conclusion
HC CA	0.378***	HC DC		0.522***	DC CA	0.215		0.112 ns	0.490***	22.893	[ − 0.117, 0.121]	0.956	Direct-only nonmediation
OL CA	0.365***	OL DC		0.598***	DC CA	0.213		0.127 ns	0.492***	25.869	[ − 0.163, 0.146]	0.958	Direct-only nonmediation
ED CA	0.464***	ED DC		0.558***	DC CA	0.112		0.062 ns	0.526***	11.870	[ − 0.158, 0.154]	0.961	Direct-only nonmediation
DM CA	0.168*	DM DC		0.365***	DC CA	0.337**		0.123*	0.291*	42.269	[ − 0.076, 0.084]	0.929	Complementary mediation

*CA* Competitive advantages; *DC* Dynamic capabilities; *DM* Digital marketing; *ED* Environmental dynamism; *HC* Human capital; *OL* Organizational learning; *VAF* Variance accounted For. ***, **, and * denote *p* < 0.001, 0.01, and 0.05 respectively

## Discussion

### Theoretical contributions

By successfully operationalizing the measurement model for dynamic capabilities as a second-order construct, this study addresses the challenge facing firms within the tourism context. This result contributes to the theory of dynamic capabilities and the relevant framework by Teece and Pisano (1994). Previous studies usually measure dynamic capabilities as a single-dimensional construct (Hawass 2010; Verreynne et al. 2016) even though, by nature, dynamic capabilities is a multidimensional construct (Laaksonen and Peltoniemi 2018; Nyamrunda and Freeman 2021).

Furthermore, this study has investigated the antecedents and outcomes of dynamic capability both internally and externally at the organizational level, namely, at the firm level. In contrast to existing literature that predominantly focuses on the dynamic capabilities of manufacturing and hi-technology businesses, this study draws from the dynamic capabilities view as the theoretical framework to provide new insights into which factors and how they contribute to the dynamic capabilities of a key service – tourism – at the level of the firm.

Our results demonstrate that dynamic capabilities in tourism firms are influenced primarily by environmental dynamism that occurs outside organizations, followed by the organizational learning culture, human capital, and the application of digital marketing within the organization. This significance level suggests that organizations are alert to the importance of fluctuations in the external environment that may affect their ability to sense and seize changes and reconfigure their processes, routines, and activities to keep pace with external dynamism. This finding reflects the reality in Vietnam – a dynamic market–which is evaluated as one of the world's most attractive tourist destinations (World Economic Forum 2019) and predicted to be one of the most sought-after international tourist places in post-Covid-19 pandemic.^4^ Previous research by Li and Liu (2014) shows that environmental dynamism is an important determinant of dynamic capabilities. Other studies, however, do not explicitly claim that environmental dynamism is an antecedent of dynamic capabilities.

Instead, they affirm it as an important factor that influences the relationship between dynamic capabilities and organizational performance (Castiaux 2012; Drnevich and Kriauciunas 2011), provided the right dynamic capability (either sensing, seizing, or reconfiguring) is applied appropriately in line with different levels of environmental dynamism (Li et al. 2019; Piening and Salge 2015; Teece 2007). A recent study by Maldonado-Guzmán et al. (2017) finds that the external environment is the barrier most likely to limit the innovative capabilities of SMEs in the service sector in Mexico. The current shock caused by the COVID-19 pandemic to economies is likely to radically affect the different capabilities of organizations to adapt and innovate to respond to the new ‘normal’ (Papadopoulos et al. 2020; Seetharaman 2020).

An organizational learning culture has been recognized as one of the key antecedents of dynamic capabilities for Vietnamese tourism organizations. Hung et al. (2010) find that dynamic capabilities mediate the influence of organizational learning culture on organizational performance and that the organizational learning culture impacts dynamic capabilities. Knowledge resources and learning mechanisms in organizations positively influence dynamic capabilities, while the learning mechanism mediates the relationship between knowledge resources and dynamic capabilities (Chien and Tsai 2012). The influence of organizational learning culture on dynamic capabilities identified in our study confirms the importance of a learning process in organizations and further enhances the scholarly discussion of the same topic in previous studies. For example, studies by Bendig et al. (2018) argue that a firm’s knowledge-based capital is part of the micro-foundation of dynamic capabilities and firm leaders indirectly influence organizational dynamic capabilities by creating individual learning conditions. The study by Linden et al. (2019) discusses the contribution of ‘knowing’ in practice to developing dynamic capabilities.

Our results confirm that the level of knowledge, skills, and abilities among employees significantly impacts the development of dynamic capabilities in Vietnamese tourism organizations. The results are consistent with Nieves and Haller's (2014) research that uses a sample of Spanish firms in the hotel industry and Kale et al.’s (2019) research about tourist accommodation establishments. Furthermore, our finding supports the strategic management literature on tourism by pointing out that micro-foundation factors (such as people) are the drivers for dynamism, advancement, progress, or improvement in organizations (Biesenthal et al. 2019; Marzo and Scarpino 2016). Thus, this research provides conclusive support for the claim by Rothaermel and Hess (2007) and more recently in the qualitative study of smaller firms in transitional economies by Nyamrunda and Freeman (2021) that investigations of the adaptation (i.e., sensing, seizing, and reconfiguring) of firms without considering human intellectual capital is inappropriate and incomplete. Our results reinforce previous theoretical work on the important role of human capital in organizations (Macher and Mowery 2009; Nieves and Haller 2014; Xing et al. 2020). As such, our results provide empirical evidence for further research on the role of micro-foundational factors (i.e., individuals) in organizations as determinants of dynamic capabilities.

The effect of digital marketing applications on dynamic capabilities is weaker than that of human capital. Prior studies suggest that technology significantly facilitates and enables a firm’s agility and adaptability (Chakravarty et al. 2013; Vogel and Güttel 2013). Our finding shows the contrast for tourism firms. It is the human factor that facilitates dynamic capabilities, regardless of which technology is used in the firm. Nevertheless, our results coincide with those of Singh and Rao (2016), Biesenthal et al. (2019), and Nyamrunda and Freeman (2021), who find that intellectual capital has a strong effect on dynamic capabilities and contributes significantly to the integration and reconfiguration of such capabilities.

This study validates the significant relationship between dynamic capabilities and an organization's competitive advantage. The results show a consistently positive impact of dynamic capabilities on competitive advantage. Previous studies find a similar linkage between dynamic capabilities and outstanding performance of organizations (Ferreira et al. 2020; Ojha et al. 2020; Ringov 2017). The outcomes of this research and the studies mentioned above collectively reinforce the ideas of Teece (2014) that dynamic capabilities do not operate alone and must be combined with effective strategizing to yield a competitive advantage. Dynamic capabilities are thus a source of competitive advantage (Salvato and Vassolo 2018) and affect the performance of organizations through the influence of different levels of environmental dynamism (Protogerou et al. 2012). Schilke’s (2014) mixed-methods study shows that dynamic capabilities are associated with a competitive advantage in moderately dynamic rather than stable or highly dynamic environments. Schilke (2014) demonstrates that the level of external dynamism influences this relationship. The positive influence dynamic capabilities exert on organizational performance forms part of the literature on organizational capabilities, demonstrating that it is a critical source of organizational performance (Wernerfelt 1984). With competitive advantage as the source of superior performance (Porter 1985), the results of this study indicate that the relationship between dynamic capabilities and organizational performance also contributes to the literature on organizational capabilities and performance. Strong dynamic capabilities must be integrated with a good strategy to achieve substantial performance (Teece 2014).

The mediation analyses confirm no mediating effects of dynamic capabilities on the direct relationship between human capital and competitive advantage, organizational learning and competitive advantage, and environmental dynamism and competitive advantage. However, as Table 8 indicates, although the indirect effects between these antecedents and competitive advantage are insignificant, the total effect of this relationship is significant. Thus, the mediation effect of dynamic capabilities exists but is too mild (as can be seen from the VAF values) to produce a significant relationship between the first three antecedents and competitive advantage. This suggests sufficient statistical power to detect the full effect but not enough to detect the effect when decomposed into its parts (Loeys et al. 2015). Taken together, the mediation effects of dynamic capabilities are too small to detect.

### Managerial implications

Our study provides useful insights to managers looking to enhance their tourism business and the development of the tourism sector. Specifically, it points to the importance of the external environment in the operations of tourism organizations. The external environmental dynamism is even more important in emerging economies like Vietnam, where the changes are intense and continuous. Additionally, the clients’ demands in a developing country for new products and services require the volumes of products and services to be delivered to change fast and often. Therefore, managers in organizations should evaluate the environment to ensure they adopt the most suitable strategy to support their company’s operations.

Moreover, managers should build a learning culture in which people are willing to share their ideas and facilitate the learning process. This action may enhance the organization’s capabilities to change and cope with new developments in the market. In conjunction with improving the quality of new recruits, creating a positive learning culture within organizations will ensure that tourism companies have better quality human capital, thus supporting their sustained success.

Furthermore, in terms of the low influence of digital marketing on dynamic capabilities found in this study, the costly investment to digitalization for both SMEs and large tourism firms should be questioned. Even though digital marketing was ranked fourth in the order of influence on dynamic capabilities, managers in tourism firms need to expend considerably more effort in improving their cyberspace presence to attract more customers. The implication for the application of digital marketing is becoming more important, given the latest movement to more digitalization in the tourism sector and changes in tourist behavior for safety because of COVID-19 (Talwar et al. 2022).

### Limitations and future research directions

Some limitations of this study have implications for future research. First, various control variables might have been omitted that significantly influence competitive advantages. Therefore, additional internal and external variables influencing the deployment and performance of dynamic capabilities could be included. Similarly, further work is required on the strength of each capability (influenced by each determinant). Second, our data are cross-sectional and collected in a pre-defined period. This means the results might be limited in the extent to which they reflect the influence of dynamic capabilities on long-term performance and sustained competitive advantage over time. With the shocks and disruptions caused by the ongoing COVID-19 pandemic to all economies, more extensive and longitudinal research will be important to evaluate how dynamic capabilities can be sustained during a time of crisis. Third, the responses obtained might have been subjective to individuals at the point of collecting data. It is thus possible that different results would have been attained had the data been collected at a different time. Finally, due to the limited capacity of existing software that cannot calculate the R-squared of a non-recursive model like ours, this paper is incapable of providing R-squared values for the calculated models. Instead, we report the key fit indexes that have been widely suggested and applied multivariate data analysis literature (e.g., Hair et al. 2014 and Mai et al. 2021).

### Electronic supplementary material

Below is the link to the electronic supplementary material.

Supplementary file1 (DOCX 22 KB)

Supplementary file2 (DOCX 180 KB)

## Appendix 1

### Extracted constructs from the questionnaire

#### Dynamic capabilities (DC)

##### Sensing (SS)

**Table:**

SS1	We frequently scan the macro-environment (the national economy, information, and technology, population, demography) to identify new business opportunities
SS2	We frequently scan the microenvironment (laws in tourism, infrastructure for tourism, skills of labour in the tourism sector, investment scale and business capacity of tourism firms) to identify new business opportunities
SS3	We regularly evaluate the possible impact of changes in our business environment on customers
SS4	We often review our service development efforts to ensure they are in line with what customers want
SS5	We spent a lot of time implementing ideas for new tourism services
SS6	We spent a lot of time improving our existing tourism services

##### Seizing (SZ)

**Table:**

SZ1	We invest in finding solutions for our customers
SZ2	We adopt the best practices in our tourism sector
SZ3	We respond to weaknesses pointed out by employees
SZ4	We change our practices when customer feedback gives us a reason to change

##### Reconfiguring (RCFG)

**Table:**

RCFG1	We annually implement new management methods
RCFG2	We annually change our marketing strategy
RCFG3	We annually renew business processes
RCFG4	We constantly renew the ways of achieving our goals

##### Human capital (HC)

**Table:**

HC1	Our employees have high working skills
HC2	Our employees are creative
HC3	Our employees are experts in their jobs
HC4	Our employees develop new ideas

##### Organizational learning (OL)

**Table:**

OL1	My company communicates lessons learned to all employees
OL2	My organization gives people choices in their work assignments
OL3	My company gives people control over the resources they need to accomplish their work
OL4	My organization encourages people to get their answers from across the organization when solving problems
OL5	In my company, leaders generally support requests for learning opportunities and training
OL6	In my organization, leaders mentor and coach those they lead

##### Digital marketing

**Table:**

DM1	Promote and advertise company’s products, services, and capabilities
DM2	Provide online product catalogue to customers and prospective customers
DM3	Answer customer queries about product and service availability, booking status, among other aspects
DM4	Allow customers to book our services online
DM5	Enable salespeople to have online access to product, price, and performance information
DM6	Enable salespeople to transmit sales call information online
DM7	Enable online purchase of products and services from suppliers
DM8	Provide online support to travel agencies

##### Environmental dynamism

**Table:**

ED1	Environmental changes in our local market are intense
ED2	Our clients regularly ask for new products and services
ED3	In our local market, changes are taking place continuously
ED4	In our market, the volumes of products and services to be delivered change fast and often

##### Competitive advantage

**Table:**

CA1	Our company has more strategic advantages than our competitors
CA2	Our company has a large market share
CA3	Our company is more successful than our main competitors are
CA4	Our EBIT (earnings before interest and taxes) is continuously above the industry average
CA5	Our ROI (return on investment) is continuously above the industry average
CA6	Our ROS (return on sales) is continuously above the industry average

## Footnotes

1 https://vietnamtimes.org.vn/vietnam-remains-an-attractive-destination-for-international-visitors-40300.html.

2 According to Hair et al. (2014, p. 115), a good rule of thumb is that factor loading estimates should be 0.5 or higher, and ideally 0.7 or higher.

3 Bootstrapping is a statistical technique that is built on repeated sampling with replacement from an initially given sample of raw data (Efron and Tibshiriani 1986).

4 https://vietnamtourism.gov.vn/english/index.php/items/16926.
