# Supplementary material for: Dynamic capabilities in tourism businesses: antecedents and outcomes
Source: Rev Manag Sci. 2022 Jun 27;17(5):1645–80. doi: 10.1007/s11846-022-00567-z (PMC9243791; doi:10.1007/s11846-022-00567-z)
Supplement: Supplementary file 1 — Supplementary file1 (DOCX 22 KB) [file 11846_2022_567_MOESM1_ESM.docx]

**SUPPLEMENTARY DOCUMENT 1- Factor Loading Results**

| **Constructs** | **Items** | **Code** | **Factor loadings** |
| --- | --- | --- | --- |
| Sensing | We frequently scan the macro environment (the national economy, information and technology, population, demography) to identify new business opportunities. | **SS1** | .65 |
|  | We frequently scan the microenvironment (laws in tourism, infrastructure for tourism, skills of labour in tourism sector, investment scale and business capacity of tourism businesses) to identify new business opportunities. | **SS2** | .69 |
|  | We periodically review the likely effect of business environment’s changes on customer demand. | **SS3** | .74 |
|  | We often review our service development efforts to ensure they are in line with what customers want. | **SS4** | .79 |
|  | We spent a lot of time implementing ideas for new tourism services. | SS5^c^ | .57 |
|  | We spent a lot of time improving our existing tourism services. | SS6 ^c^ | .56 |
| Seizing | We invest in finding solutions for our customers. | **SZ1** | .70 |
|  | We adopt the best practices in our tourism sector. | SZ2 | N/A |
|  | We respond to weaknesses pointed out by employees. | **SZ3** | .78 |
|  | We change our practices when customer feedback gives us a reason to change. | **SZ4** | .64 |
| Reconfiguring | We annually implement new management methods. | RCFG1^a^ | .65 |
|  | We annually change our marketing strategy. | **RCFG2** | .75 |
|  | We annually renew business processes. | **RCFG3** | .77 |
|  | We constantly renew the ways of achieving our goals. | **RCFG4** | .84 |
| Human Capital | Our employees have high working skills. | **HC1** | .69 |
|  | Our employees are creative. | **HC2** | .76 |
|  | Our employees are experts in their jobs. | **HC3** | .85 |
|  | Our employees develop new ideas. | **HC4** | .71 |
| Organisational Learning | Our organisation makes its lessons learned available to all employees. | **OL1** | .75 |

(Table continued on the next page)

| Organisational Learning  (continued) | Our organisation gives people choices in their work assignments. | **OL2** | .70 |
| --- | --- | --- | --- |
|  | Our organisation gives people control over the resources they need to accomplish their work. | OL3 ^c^ | .52 |
|  | Our organisation encourages people to consult across the organisation when solving problems. | **OL4** | .70 |
|  | In our organisation, leaders generally support requests for learning and training opportunities. | OL5^a^ | .65 |
|  | In our organisation, leaders mentor and coach those they lead. | OL6 ^c^ | .59 |
| Environmental Dynamism | Environmental changes in our target are intense. | ED1^a^ | .62 |
|  | Our clients regularly ask for new products and services. | **ED2** | .74 |
|  | In our target market, changes in tastes and demands are taking continuously. | **ED3** | .76 |
|  | In our target market, the volumes of products and services to be delivered change fast and often. | **ED4** | .72 |
| Digital Marketing | Promote and advertise company’s products, services, and capabilities. | DM1^c^ | .56 |
|  | Provide online product catalogue to customers and prospective customers. | DM2^c^ | .58 |
|  | Answer customer queries about product and service availability, booking status, among other aspects. | **DM3** | .67 |
|  | Allow customers to book our services online. | **DM4** | .67 |
|  | Enable sales people to have online access to product, price, and performance information. | **DM5** | .81 |
|  | Enable salespeople to transmit sales call information online. | **DM6** | .83 |
|  | Enable online purchase of products and services from suppliers. | DM7^a^ | .84 |
|  | Provide online support to travel agencies. | **DM8** | .71 |
| Competitive Advantage | We have gained strategic advantages over our competitors. | **CA1** | .77 |
|  | We have a large market share. | **CA2** | .86 |
|  | Overall, we are more successful than our major competitors are. | **CA3** | .89 |
|  | Our EBIT (earnings before interest and taxes) is continuously above industry average. | **CA4** | .72 |
|  | Our ROI (return on investment) is continuously above industry average. | CA5^b^ | .85 |
|  | Our ROS (return on sales) is continuously above industry average. | **CA6** | .80 |

**Notes**

The items in **bold code** are the final measurement items for the statistical model.

^a^: Removed because of large standardized residual covariance^[[1]](#footnote-1)^ (larger than |2.58| (Byrne, 2010: 77)

^b^: highly correlated with CA4 and CA6 at the same time.

^c:^ Factor loadings lower than 0.6

N/A: the item was removed before distributing the questionnaire as per experts’ suggestions

1. Residual covariances: The difference between the sample covariance and the covariance expected under the fitted model provide a natural estimate of the fit of covariance structure models; the larger (in absolute value) the residual covariance, the worse the fit (Maydeu-Olivares & Shi, 2017: 23). [↑](#footnote-ref-1)
