# Supplementary figures and images for: Dynamic capabilities in tourism businesses: antecedents and outcomes
Source: Rev Manag Sci. 2022 Jun 27;17(5):1645–80. doi: 10.1007/s11846-022-00567-z (PMC9243791; doi:10.1007/s11846-022-00567-z)

**CFA Model with Results**


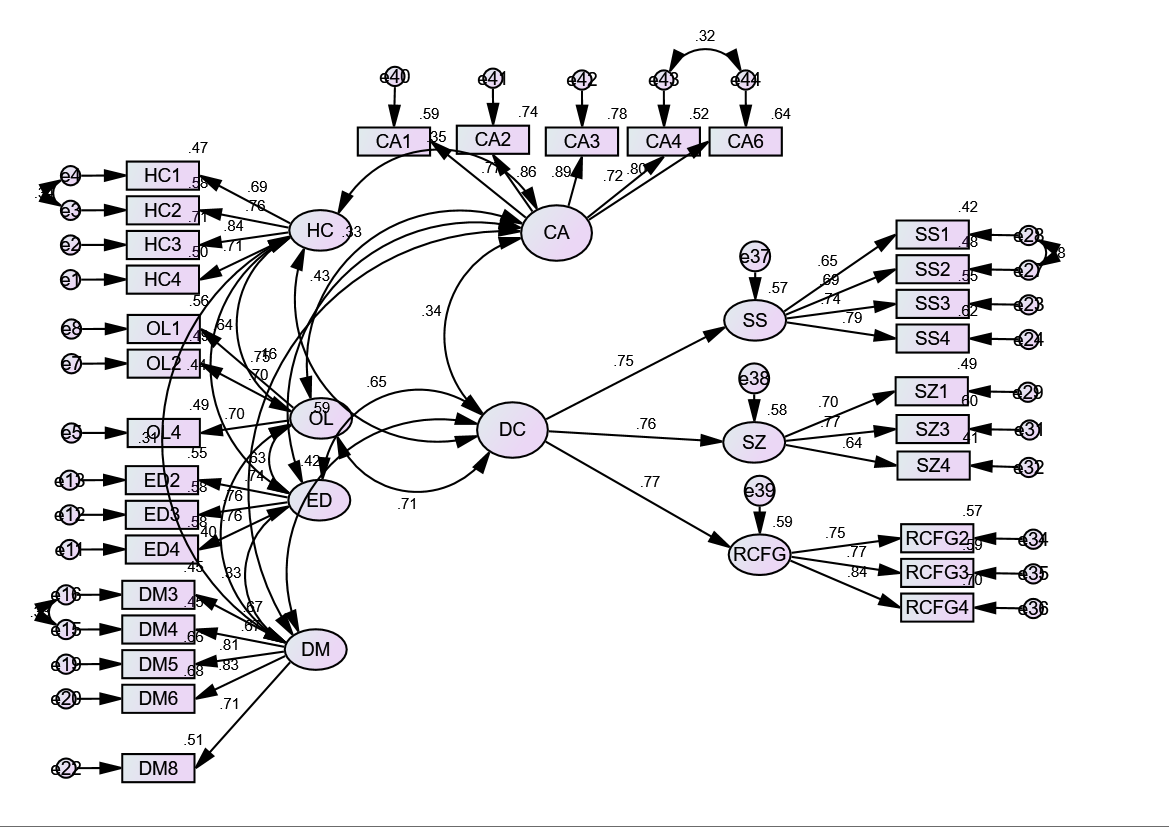

Supplement: Supplementary file 2 — Supplementary file2 (DOCX 180 KB) [file 11846_2022_567_MOESM2_ESM.docx]
